# Mixed Infection in Common Carp (*Cyprinus carpio*) Caused by *Aeromonas veronii*, *Aeromonas hydrophila*, *Plesiomonas shigelloides*, and *Citrobacter freundii*

**DOI:** 10.3390/ani15060805

**Published:** 2025-03-12

**Authors:** Jinghang Zhang, Dan Qiao, Haoyu Wang, Xianliang Zhao, Xinyu Jiang, Lei Zhu, Jie Zhang, Li Li, Xianghui Kong, Chao Pei

**Affiliations:** Engineering Lab of Henan Province for Aquatic Animal Disease Control, College of Fisheries, Henan Normal University, Xinxiang 453007, China; 2220283041@stu.htu.edu.cn (J.Z.); qiaodan0308@126.com (D.Q.); 15093406242@163.com (H.W.); zxl830724@163.com (X.Z.); jiangxinyu@htu.edu.cn (X.J.); zhulei@htu.edu.cn (L.Z.); 2016030@htu.edu.cn (J.Z.); lily-fish@htu.edu.cn (L.L.); xhkong@htu.edu.cn (X.K.)

**Keywords:** pathogenic bacteria, challenge experiment, opportunistic infection, antibiotic resistance

## Abstract

*Aeromonas veronii*, *Aeromonas hydrophila*, *Plesiomonas shigelloides*, and *Citrobacter freundii* are opportunistic pathogens that are widely distributed in aquatic environments and fish populations, causing fish diseases under stressful conditions. In this study, bacteria were isolated from a group of diseased common carp, *Cyprinus carpio*, with symptoms of hemorrhaging along the body surface, abdominal distention, and flatulence in the intestine. According to the results of biochemical characteristics and *16S rRNA* sequencing analyses, the isolates were identified as *A. veronii*, *A. hydrophila*, *P. shigelloides*, and *C. freundii*. The virulence genes from these four representative bacterial strains were amplified. The experimentally co-infected fish showed similar symptoms to those observed in the naturally infected common carp. The LD_50_ of *C*. *freundii*, *P*. *shigelloides*, *A. veronii*, *A. hydrophila*, and the mixture group were 1.95 × 10^4^, 4.74 × 10^4^, 5.12 × 10^4^, 1.53 × 10^5^, and 5.41 × 10^4^ CFU, respectively. The antibiotic resistance results showed that *P*. *shigelloides*, *A. veronii*, and *A. hydrophila* were most sensitive to ceftizoxime, florfenicol, enrofloxacin, and norfloxacin. *C*. *freundii* was most sensitive to ceftizoxime and gentamicin.

## Simple Summary

*Aeromonas veronii*, *Aeromonas hydrophila*, *Plesiomonas shigelloides*, and *Citrobacter freundii* are opportunistic pathogens and cause fish diseases in aquaculture. The four kinds of bacteria were isolated from a group of diseased common carp. Artificial infection indicated that the four isolates were all highly pathogenic to fish. Similar cases of mixed bacterial infection in farmed common carp have rarely been reported previously, and our study on bacterial drug resistance provides a reference for treatment of bacterial fish diseases caused by these pathogens.

## 1. Introduction

The common carp (*Cyprinus carpio*) is a fish that is cultured worldwide and is also one of the most important economic fish species in China [1]. The total production of farmed *C. carpio* was 2.87 million tons in 2023, according to the China Fishery Statistical Yearbook. However, improper management and high-density culture create the ideal conditions for explosive epidemics of various diseases. It has been reported that the *C. carpio* breeding industry is threatened by a variety of bacterial organisms [2,3,4,5]. *Aeromonas veronii* is a Gram-negative bacterium that is widely distributed in aquatic environments and can cause ulcerative diseases in many fishes [6,7,8,9]. The main clinical symptoms of *A. veronii* infection are hemorrhagic septicemia, fin rot, exophthalmia, and abdominal distention [9]. *A. hydrophila* is another *Aeromonas* species that is highly similar to *A. veronii* in pathogenicity [10]. *Plesiomonas shigelloides* and *Citrobacter freundii* both belong to the Enterobacteriaceae family. *P. shigelloides* has been reported to cause protruding eyeballs, swollen anus, ascites in the abdominal cavity, swelling, and hemorrhaging in diseased fish [11,12]. *C. freundii* infection is associated with enteritis, necrosis, body reddening, hemorrhaging, and septicemia [13,14,15,16]. The four species of bacteria are all opportunistic pathogens and can be commonly found in normal aquaculture water and healthy fish. They are more likely to cause diseases when fish are exposed to various stressors, such as temperature change, hypoxia, and parasite infection [17,18].

Common carp disease has historically been attributed to many bacteria. However, the mixed infection of *A. veronii*, *A. hydrophila*, *P. shigelloides*, and *C. freundii* in common carp has not been reported before. Research into the pathogenicity and drug resistance of these four bacteria would help to provide a reference for the prevention and treatment of similar cases.

## 2. Materials and Methods

### 2.1. Fish

The diseased common carp (*n* = 46, 18.3 ± 2.6 g) were obtained from the aquaculture base of Henan Normal University in Xinxiang city, Henan province, China. The fish were kept in a concrete pond (30 m^2^) with a water depth of 1.5 m and at a water temperature of approximately 32 °C. Moribund fish were transported immediately to the laboratory for diagnosis and pathogen isolation. For the experimental infection, healthy common carp (16 ± 2.1 g) with no history of disease were obtained from a local fish farm in Xinxiang city. The healthy fish were acclimated in aquaria (200 L each) for two weeks prior to the infection assay and fed with commercial feed at 1% of their body weight once daily. Half of the total volume of water was replaced daily, and the water temperature was maintained at 28 ± 1 °C. The experiments involving live fish were conducted in accordance with the American National Research Council’s “Guide for the Care and Use of Laboratory Animals”.

### 2.2. Isolation and Identification of Bacteria

Ten bacterial strains were isolated from the ascites of moribund fish and identified through the *16S rRNA* genes, following the method used in previous reports [7]. The biochemical characteristics were examined using a commercial reagent (Hangzhou Microorganism Reagent Co., Ltd., Hangzhou, China). The 10 strains identified were intraperitoneally injected into healthy fish (10 fish each strain) at 1.0 × 10^5^ colony-forming units (CFU) per fish. The highest mortality strain in each bacterial species, namely *A. hydrophila* XX236, *A. veronii* XX237, *C. freundii* XX238, and *P. shigelloides* XX239, was chosen as a representative strain of each species and used for the following studies (Table S1). The primers used to detect the virulence genes were designed according to the previous reports [7,12,14,19]. The amplified virulence genes were verified by sequencing.

### 2.3. Antibiotic Susceptibility Testing

The antibiotic susceptibility of aeromonad was determined by the Kirby–Bauer disk diffusion method [20]. The bacterial strains were streaked on Mueller–Hinton agar plates, and the various antibiotic disks (Difco Laboratories, Detroit, MI, USA) were applied on the streaked cultures. After 18 h of incubation at 28 °C, the sizes of the zone of bacterial growth inhibition were measured. The isolates were classified as sensitive (S), moderately sensitive (M), or resistant (R) according to the National Committee for Clinical Laboratory Standards.

### 2.4. Experimental Infections

For median lethal dosage (LD_50_) determination, 210 carp individuals were randomly divided into six groups, including five infected groups (40 fish per group) and one control group (10 fish). The infected groups were intraperitoneally injected with *A. veronii*, *A. hydrophila*, *P*. *shigelloides*, *C*. *freundii*, and a mixture (equal colony numbers of each bacterial species) at the concentrations of 1.0 × 10^4^, 1.0 × 10^5^, 1.0 × 10^6^, and 1.0 × 10^7^ CFU/fish (10 fish each), respectively. All the bacterial strains were grown in LB medium for 24 h at 28 °C before use. The control group was injected with the same dose of sterile physiological saline. The mortality and clinical signs of all groups were recorded every day for 15 d post-infection. The LD_50_ was calculated based on the total cumulative mortality (%) as described by Reed and Muench (1938) [7]. Moribund fish underwent routine bacteriological examination to re-isolate and re-identify the present organisms.

## 3. Results

### 3.1. Clinical Signs and Isolation of Bacteria from Diseased Common Carp

**Figure 1:**
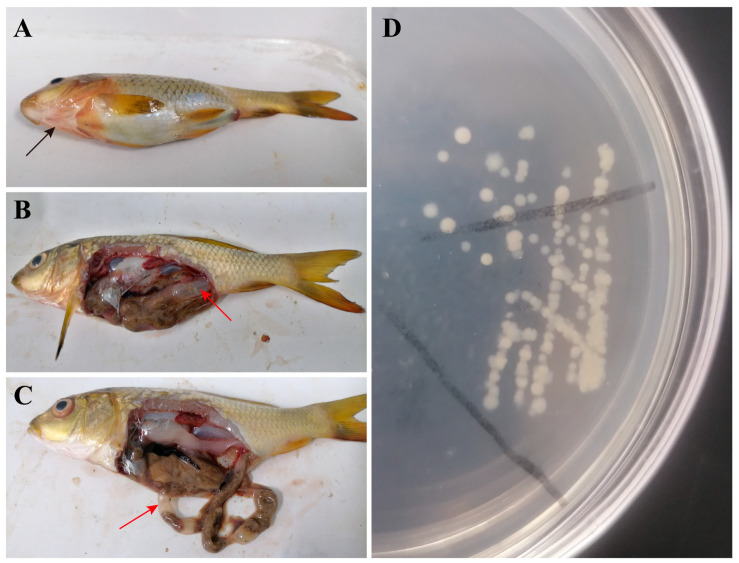
Symptoms of naturally infected fish (**left**) and colonies on LB plate (**right**). (**A**) The external symptoms of diseased fish. (**B**,**C**) Symptoms of internal organs. (**D**) Colonies on LB plate. Black arrow shows hemorrhaging along the body surface, and red arrow shows the flatulence in intestine.

All of the diseased fish showed hemorrhaging along the gill cover and lower jaw, extended abdomen, swollen anus (Figure 1A), ascites, and intestinal flatulence (Figure 1B,C). All the colonies were smooth-edged, circular, and translucent buff or cream in color (Figure 1D). Ten colonies were selected, and the *16S rRNA* were obtained and sequenced. The biochemical characteristics of the ten bacterial strains were also tested, and the results indicated the presence of the representative strains (Table S1). *A. veronii* (five strains), *P*. *shigelloides* (two strains), *C*. *freundii* (two strains), and *A. hydrophila* (one strain) were detected.

### 3.2. Phylogenetic Tree and Virulence Related Genes

Based on the *16s rRNA* sequences of *A. hydrophila* XX236, *A. veronii* XX237, *C. freundii* XX238, *and P. shigelloides* XX239, a phylogenetic tree was constructed to elucidate the genetic relationships among the four isolates and the other representative species. The isolates of XX236, XX237, XX238, XX239 were grouped with *A. hydrophila*, *A. veronii*, *C. freundii*, and *P. shigelloides*, respectively (Figure S1).

**Figure 2:**
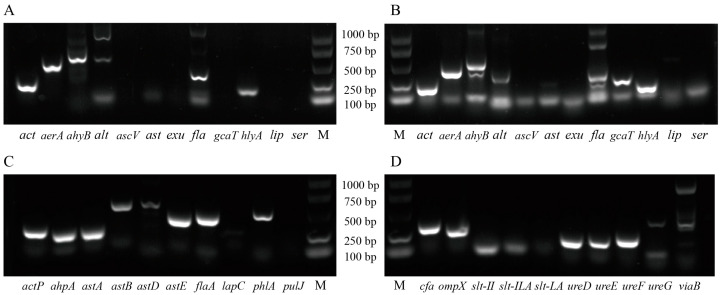
Detection of virulence genes in *A. hydrophila* (**A**), *A. veronii* (**B**), *P. shigelloides* (**C**), and *C. freundii* (**D**). M: Maker.

For the virulence gene detection, *act*, *aerA*, *ahyB*, *fla*, and *hlyA* were detected in *A. hydrophila. Act*, *aerA*, *ahyB*, *alt*, *fla*, *gcaT*, and *hlyA* were present in *A. veronii. P. shigelloides* had *actP*, *ahpA*, *astA*, *astB*, *astD*, *astE*, *flaA*, and *phlA* genes, while *C. freundii* was positive for the *cfa*, *ompX*, *ureD*, *ureE*, *ureF*, and *viaB* genes (Figure 2).

### 3.3. Experimental Infections

**Figure 3:**
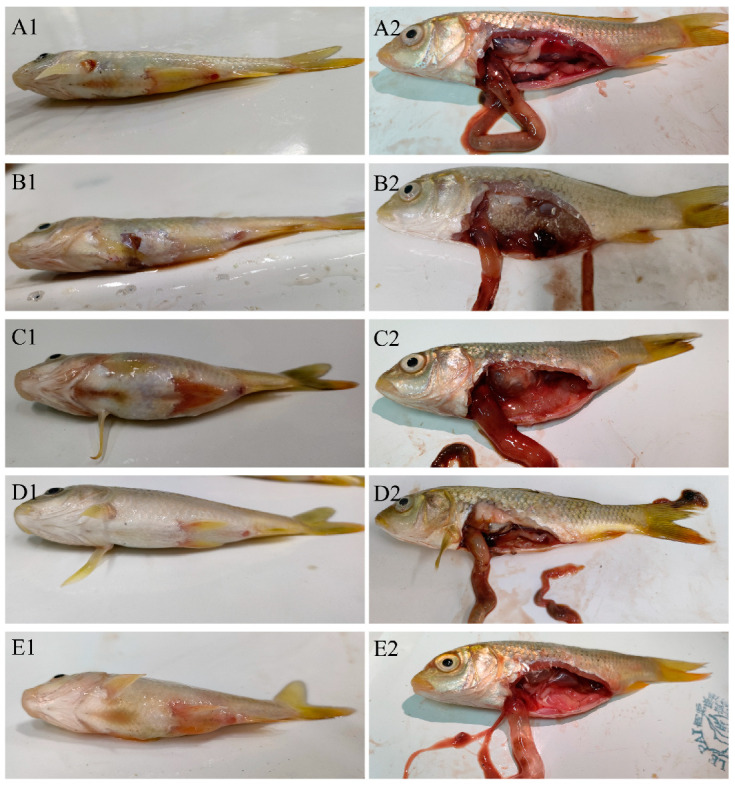
Symptoms on the epithelial surface and internal organs of experimentally infected fish. (**A1**,**A2**), *A. veronii* infection, (**B1**,**B2**), *A. hydrophila* infection, (**C1**,**C2**), *P. shigelloides* infection, (**D1**,**D2**), *C. freundii* infection, (**E1**,**E2**), mixed infection.

To confirm the pathogenicity of the representative strains, a challenge assay was carried out in the healthy common carp. The fish were intraperitoneally injected with different doses of each bacterial strain or a mixture. The fish injected with *Aeromonas* (Figure 3(A1,B1)) had protruding eyes, ulceration on the abdomen, and redness of the anus. The *P*. *shigelloides*-infected fish showed an extended and ulcerated abdomen, as well as a fin hemorrhage (Figure 3(C1)). Fin hemorrhage was also observed in the *C*. *freundii*-infected fish (Figure 3(D1)). The mixed infection group had all the above symptoms (Figure 3(E1)). All the artificially infected fish showed ascites and flatulence in the intestine (Figure 3(A2–E2)). The LD_50_ of *C*. *freundii*, *P*. *shigelloides*, *A. veronii*, and *A. hydrophila* were 1.95 × 10^4^, 4.74 × 10^4^, 5.12 × 10^4^, and 1.53 × 10^5^ CFU, respectively. The LD_50_ of the mixture group was 5.41 × 10^4^ CFU (Table 1). Furthermore, the same bacterial species were re-isolated from the experimentally infected fish, as confirmed by colonial morphology observation, physiological and biochemical characteristics analyses, and *16S rRNA* sequencing analyses.

### 3.4. Determination of Antimicrobial Resistance

The antibiotic resistance patterns of the four bacteria, measured by the size of the inhibition zones around each disk, showed that *P*. *shigelloides*, *A. veronii*, and *A. hydrophila* were sensitive to streptomycin, enrofloxacin, florfenicol, gentamicin, kanamycin, neomycin, norfloxacin, co-trimoxazole, ceftizoxime, and resistant to ampicillin. *C*. *freundii* was sensitive to streptomycin, gentamicin, and ceftizoxime, moderately susceptible to kanamycin, neomycin, norfloxacin, and ampicillin, and resistant to enrofloxacin, florfenicol, tetracycline, and co-trimoxazole (Table 2).

## 4. Discussion

The co-infection cases of two (*C. freundii* and *P. shigelloides*) or three (*A. bestiarum*, *A. sobria*, and *P. shigelloides*) bacteria in fish have been reported [17,21]; however, we report, for the first time, on a mixed infection with four bacterial species (*A. veronii*, *A. hydrophila*, *C. freundii*, and *P. shigelloides*) in common carp. The phylogenetic tree analysis further confirmed the identification of those strains besides the biological characterization. The experimental infections showed that fish injected with the different bacterial strains developed varying clinical symptoms (Figure 3(A1–D2)), but the artificially co-infected fish showed similar symptoms with the naturally infected fish, which were hemorrhaging, abdominal distention, ascites, and flatulence (Figure 1 and Figure 3(E1,E2)), indicating that the clinical signs in observed in the naturally infected fish may be a result of co-infection. However, it was difficult to identify which bacteria was the main cause of the disease by the symptoms alone. But since half of the ten isolated strains were *A. veronii*, and the LD_50_ of mixture group (5.41 × 10^4^ CFU) was close to that of the *A. veronii* group (5.12 × 10^4^ CFU), it is most likely that *A. veronii* was the main pathogen. The clinical symptoms of ulceration, redness of the anus, intestinal hyperemia, and flatulence commonly caused by *A. veronii* were also the main symptoms in the co-infected fish.

Virulence genes are important contributors to the pathogenicity of bacteria [7,12,22]. The high pathogenicity of *A. hydrophila*, *A. veronii*, *C. freundii*, and *P. shigelloides* may be associated with the virulence-related gene families that we detected (*act*, *aer*, *ahp*, *ahy*, *alt*, *ast*, *fla*, *gca*, *hly*, *phl*, *cfa*, *omp*, and *ure*). The LD_50_ determination showed that *C*. *freundii* was the most virulent bacteria, and *A. hydrophila* was the weakest. The virulence of the mixed bacteria group was close to the average amount (Table 1).

The results of the antimicrobial susceptibility testing indicated that *P. shigelloides*, *A. veronii*, and *A. hydrophila* were not significantly different from those previously reported [22]. However, the results for *C. freundii* differed significantly from the other fish species [14,18]. The susceptibility test is essential for the selection of drugs for treatment.

## 5. Conclusions

In this study, we isolated *A. veronii*, *A. hydrophila*, *C. freundii*, and *P. shigelloides* from diseased common carp. The virulence genes in four bacteria were determined. Artificial infection in fish showed four strains of bacteria were all highly pathogenic and could cause the disease with hemorrhage and abdominal distention syndrome, indicating the naturally infected fish maybe died of mixed infection. The drug sensitivity of the bacteria was also tested. The findings in this study will provide reference for the diagnosis, treatment, and prevention of the disease caused by mixed bacterial infection.

## Figures and Tables

**Table 1:** Cumulative mortality of experimentally infected fish by bacterial isolates.

Group	Concentration (CFU)	Fish	Dead Fish Number on Day After Challenge															Accumulative Mortality	LD_50_ Value (CFU)
			1	2	3	4	5	6	7	8	9	10	11	12	13	14	15		
Control	0	10	0	0	0	0	0	0	0	0	0	0	0	0	0	0	0	0%	
*P. shigelloides*	1.0 × 10^7^	10	0	0	0	0	4	1	1	3	1	0	0	0	0	0	0	100%	4.74 × 10^4^
	1.0 × 10^6^	10	0	0	0	0	2	1	0	3	0	2	0	0	1	0	0	90%	
	1.0 × 10^5^	10	0	0	0	0	0	0	0	3	1	2	0	1	0	0	0	70%	
	1.0 × 10^4^	10	0	0	0	0	0	0	0	1	0	0	0	1	0	0	0	20%	
*C. freundii*	1.0 × 10^7^	10	0	0	0	0	4	2	3	1	0	0	0	0	0	0	0	100%	1.95 × 10^4^
	1.0 × 10^6^	10	0	0	0	0	1	1	2	3	1	1	1	0	0	0	0	100%	
	1.0 × 10^5^	10	0	0	0	0	0	1	0	1	5	1	1	0	0	0	0	90%	
	1.0 × 10^4^	10	0	0	0	0	0	0	0	0	3	0	0	0	0	0	0	30%	
*A. veronii*	1.0 × 10^7^	10	2	4	3	0	0	0	0	0	0	0	0	0	0	0	0	90%	5.12 × 10^4^
	1.0 × 10^6^	10	1	0	0	0	4	2	0	1	0	0	0	1	0	0	0	90%	
	1.0 × 10^5^	10	1	0	0	0	0	1	0	2	1	0	0	2	0	0	0	70%	
	1.0 × 10^4^	10	0	0	0	0	0	1	0	0	1	0	0	0	0	0	0	20%	
*A. hydrophila*	1.0 × 10^7^	10	1	0	0	0	3	1	2	0	0	1	0	2	0	0	0	100%	1.53 × 10^5^
	1.0 × 10^6^	10	0	0	0	0	1	0	1	1	4	0	1	0	0	0	0	80%	
	1.0 × 10^5^	10	0	0	0	0	0	0	0	0	1	0	1	2	0	0	0	40%	
	1.0 × 10^4^	10	0	0	0	0	0	0	0	0	0	0	0	1	0	0	0	10%	
Mixed infection	1.0 × 10^7^	10	0	0	0	0	3	1	1	2	2	1	0	0	0	0	0	100%	5.41 × 10^4^
	1.0 × 10^6^	10	0	0	0	0	1	1	0	1	2	1	2	0	1	0	0	90%	
	1.0 × 10^5^	10	0	0	0	0	1	0	0	1	0	0	1	1	0	1	0	50%	
	1.0 × 10^4^	10	0	0	0	0	0	0	0	0	0	1	2	0	0	0	0	30%	

**Table 2:** Susceptibility of bacterial isolates to antibiotics.

Antibiotic	Drug Concentration (μg/disc)	Inhibition Zone Diameter (mm)			
		*A. veronii*	*A. hydrophila*	*C. freundii*	*P. shigelloides*
Streptomycin	10	19 ^S^	15 ^S^	15 ^S^	15 ^S^
Enrofloxacin	10	40 ^S^	23 ^S^	10 ^R^	30 ^S^
Florfenicol	30	30 ^S^	32 ^S^	0 ^R^	28 ^S^
Gentamicin	10	21 ^S^	19 ^S^	20 ^S^	19 ^S^
Kanamycin	30	20 ^S^	20 ^S^	15 ^I^	18 ^S^
Neomycin	30	17 ^S^	18 ^S^	17 ^I^	18 ^S^
Tetracycline	30	11 ^R^	25 ^S^	0 ^R^	21 ^S^
Norfloxacin	10	34 ^S^	23 ^S^	14 ^I^	27 ^S^
Co-trimoxazole	23.75/1.25	19 ^S^	20 ^S^	0 ^R^	13 ^I^
Ceftizoxime	30	44 ^S^	40 ^S^	32 ^S^	37 ^S^
Ampicillin	10	10 ^R^	10 ^R^	14 ^I^	0 ^R^

Note: “^S^” is sensitive, “^I^” is intermediate, and “^R^” is resistant.

## Data Availability

Data will be made available by the authors upon request.

## Supplementary Materials

The following supporting information can be downloaded at: https://www.mdpi.com/article/10.3390/ani15060805/s1, Table S1. Mortality rate of isolated strains. Table S2. Biochemical characteristics of isolated strains. Figure S1. Neighbor-joining tree based on 16S rRNA sequence Numbers at nodes indicate bootstrap percentages derived from 1000 replications made by MEGA7 [23], numbers at the end of bacteria species names denote the Genbank accession number. Red triangles indicate the strains of present study. Reference [23] are cited in the supplementary materials.
