# Supplementary material for: Mixed Infection in Common Carp (Cyprinus carpio) Caused by Aeromonas veronii, Aeromonas hydrophila, Plesiomonas shigelloides, and Citrobacter freundii
Source: Animals (Basel). 2025 Mar 12;15(6):805. doi: 10.3390/ani15060805 (PMC11939569; doi:10.3390/ani15060805)
Supplement: Supplementary file 1 [file animals-15-00805-s001.zip › animals-3477920-supplementary.pdf]

Supplementary Table S1. Mortality rate of isolated strains.

Strain	Fish number	Death number	Mortality rate
A. hydrophila XX236	10	5	50%
A. veronii XX237	10	8	80%
A. veronii XX240	10	6	60%
A. veronii XX241	10	7	70%
A. veronii XX242	10	7	70%
A. veronii XX243	10	8	80%
C. freundii XX238	10	9	90%
C. freundii XX244	10	8	80%
P. shigelloides XX239	10	6	60%
P. shigelloides XX245	10	5	50%

Supplementary Table S2. Biochemical characteristics of isolated strains.

Test	*P. shigelloides*	*C. freundii*	Test	*A. veronii*	*A.hydropha*
H_2S production	-	+	Glucose (gas production)	+	+
Phenylalanine deaminase	-	-	Sucrose	+	+
Gluconate	-	-	Mannose	+	+
Indole reaction	+	-	Indole reaction	+	+
Voges–Proskauer	-	-	Voges–Proskauer	+	+
Citrate	-	+	Arabinose	-	+
Motility	+	+	Arginine dihydrolase	+	+
Glucose (gas production)	-	+	Inositol	-	-
Lysine decarboxylase	+	-	Lysine	+	+
Ornithine decarboxylase	+	-	Unsalted peptone water	+	+
Raffinose	-	+	3% NaCI peptone water	+	+
Sorbitol	-	+	6% NaCI peptone water	+	+
Adonitol	-	-	8% NaCI peptone water	-	-
Xylose	-	+	10% NaCI peptone water	-	-
Urease	-	+			

Note: "+" is masculine and "-" is feminine

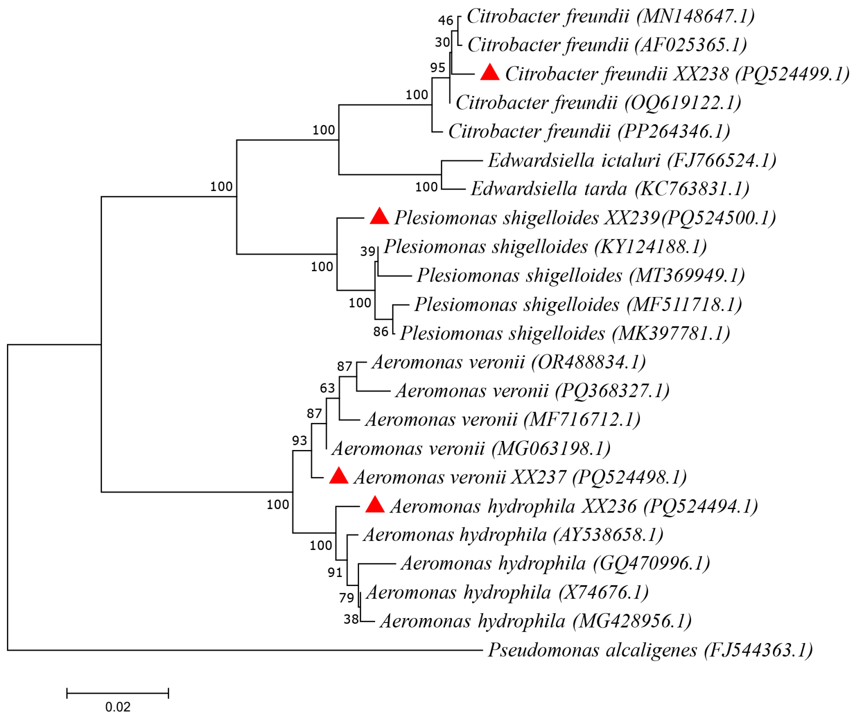

Supplementary Figure S1. Neighbor-joining tree based on 16S rRNA sequence. Numbers at nodes indicate bootstrap percentages derived from 1000 replications made by MEGA7 [23], numbers at the end of bacteria species names denote the Genbank accession number. Red triangles indicate the strains of present study.

Reference

[23] Kumar, S.; Stecher, G.; Tamura, K. MEGA7: molecular evolutionary genetics analysis version 7.0 for bigger datasets. *Mol. Biol. Evol.* **2016**, *33*, 1870-1874. https://doi: 10.1093/molbev/msw054.
